# Neoadjuvant Therapy for Esophageal Cancer

**DOI:** 10.3390/cancers18050750

**Published:** 2026-02-26

**Authors:** Nika Samadzadeh Tabrizi, Andrew Marthy, Thomas Fabian

**Affiliations:** 1 Department of Thoracic and Cardiovascular Surgery, Cleveland Clinic, Cleveland, OH 44106, USA; samadzn2@ccf.org; 2 Department of Cardiothoracic Surgery, Albany Medical Center, Albany, NY 12208, USA; marthya@amc.edu

**Keywords:** neoadjuvant, gastroesophageal cancer, chemotherapy, radiation, chemoradiation, immunotherapy

## Abstract

Background: Esophageal cancer remains one of the most aggressive and fatal malignancies worldwide. Historically, therapeutic strategies relied primarily on neoadjuvant chemotherapy or radiotherapy and surgery, but over the past two decades, randomized controlled trials have driven a major transformation in clinical practice. Methods: This narrative review synthesizes the evolution of evidence-based management from early cytotoxic regimens to modern, histology-specific treatment pathways. We examine landmark trials establishing the roles of neoadjuvant chemoradiation and chemotherapy, along with emerging data on immunotherapy—including checkpoint inhibitors. Persistent challenges include optimizing treatment selection based on molecular profiling, improving response prediction, and managing toxicity in an aging population. Conclusions: We conclude by highlighting key gaps in current evidence and outlining future directions and ongoing clinical investigations.

## Simple Summary

This review focuses on the evolving landscape of neoadjuvant therapy for esophageal cancer, highlighting current evidence, rationale, and emerging strategies. We will first examine the historical context and limitations of surgery alone, emphasizing the need for preoperative interventions. The review will discuss the role of neoadjuvant chemotherapy, chemoradiation, and immunotherapy, summarizing landmark trials as they relate to clinical practice and contemporary guidelines.

## 1. Introduction

Esophageal cancer is the 8th most common cancer globally and stands as the 6th leading cause of cancer-related mortality, accounting for approximately 400,000 deaths. Historically, squamous cell carcinoma was the most prevalent type of esophageal cancer, owing to a strong association with alcohol and tobacco use [1]. However, in developed countries, adenocarcinoma now surpasses squamous cell carcinoma, constituting roughly 80% of new esophageal cancer diagnoses. The 5-year overall survival (OS) remains poor for patients with esophageal cancer, specifically 15–20% [2]. The abysmal survival is attributed to multiple factors including lack of effective screening strategies, advanced stage at diagnosis, inadequate access to care, and therapeutic mismanagement.

In surgically resected esophageal cancer, patient survivorship rises dramatically, however recurrence occurs in nearly 50% of patients treated with surgery alone [3]. This high recurrence rate is attributed to factors such as the absence of an esophageal serosal layer, early lymphatic metastasis, and inaccuracies in preoperative staging. The aforementioned factors lead to an increased incidence of early tumor invasion, reported in almost 30% of patients with early (T1) lesions [1].

For early-stage tumors (T1a–T1b), surgical resection alone proves effective with favorable survival outcomes, approximately 60% [4]. Endoscopic mucosal resection (EMR) provides a potential option for T1a-T1b disease. However, EMR for T1b disease remains controversial due to concern for missed lymph node metastasis and higher recurrence rates [5]. Additionally, there remains debate regarding the optimal management of patients with T2N0 disease. There is evidence that neoadjuvant chemoradiation for T2N0 likely does not result in increased survival, however this fails to consider that many patients are upstaged at the time of surgery [6,7].

In cases of locally advanced disease (T3 or T4), regional lymph node (N1), or distant metastasis (M1), the 5-year OS for surgery alone is poor. To improve outcomes, neoadjuvant strategies involving chemotherapy (nCT), radiation therapy (nRT), or chemoradiation (nCRT) have been explored. Potential benefits include tumor size reduction, improved local control, facilitation of surgical resection, eradication of micro-metastases, and disease treatment. It is posited that induction therapy results in self-selection, which excludes more advanced and aggressive tumors that will progress despite surgery.

According to the 2025 National Comprehensive Cancer Network (NCCN) guidelines, neoadjuvant multimodality therapy, often involving nCRT or nCT with immunotherapy (IO), is recommended in patients with high-risk early-stage (T2 tumors ≥ 3 cm or poorly differentiated), locally advanced disease (T3-4), or nodal involvement (any N) [8]. However, there are multiple approaches to these patients and alternatives exist. The following sections aim to clarify the rationale and historical context of neoadjuvant therapy for esophageal cancer, offer specific guidance on protocols and explore emerging treatments.

## 2. Neoadjuvant Chemotherapy

The first landmark trial (RTOG 8911/American Intergroup 113) comparing nCT to surgery alone was largely negative [9,10]. It demonstrated no survival benefit with nCT, rather, R0 resection conferred the most substantial 5-year survival benefit, irrespective of whether nCT was administered. It has been suggested that the prolonged duration of nCT, as well as higher doses, contributed to nearly 20% of patients not proceeding to surgery. These findings raised concern that more intensive nCT might compromise resectability. Similarly, a trial from MD Anderson (Roth 1988) did not demonstrate a survival benefit with nCT [11]. However, the study did show improved survival among patients who responded to nCT, highlighting the prognostic significance of treatment response. Together, these results underscored the importance of tumor response to neoadjuvant therapy and complete resection, laying the groundwork for subsequent trials that have since reshaped the field.

The potential benefits of nCT are now well established. Notably, improved OS was seen in the 2002 British (MRC OE02) Trial, 2006 MAGIC Trial, and 2011 French (FNCLD/FFCD) trial [12,13,14]. According to a Cochrane review (2015) of 13 randomized controlled trials (n = 2362), nCT is linked to an overall 12% reduction in the risk of death (95% CI 0.8–0.96) and a higher rate of complete (R0) resection [15]. In addition, nCT resulted in an improved OS when compared to surgery alone. It is important to recognize that the benefit of nCT is dependent on the incorporation of adjuvant CT for adenocarcinomas. This was illustrated by EORTC 40954, which evaluated outcomes with nCT and surgery alone, with no clear survival advantage [16]. This is in contrast to the positive MAGIC and FNCLCC/FFCD trials, both of which used perioperative CT [13,14]. On the other hand, in patients with squamous cell carcinoma, the Dutch trial (2011) demonstrated a survival benefit for nCT alone [17]. Subsequent evidence has since established nCRT (without adjuvant CT or CRT) as the preferred approach for this histologic sub-type (discussed further in following section).

Survival benefit from nCT hinges on the extent of response, particularly for squamous cell carcinomas. Because pathologic complete response (pCR) rate, when followed by R0 resection, remains the strongest predictor of cure, future studies must refine patient selection to mitigate the risk of unnecessary exposure to CT and minimize toxicity in patients unlikely to benefit [18].

Despite these nuances, the superiority of nCT followed by surgery over surgery alone, regardless of histologic subtype, was redemonstrated by a landmark individual participant data network meta-analysis of 12 randomized trials involving approximately 2600 patients by Faron et al. (2023) [19].

Table 1 provides details of landmark trials comparing CT versus surgery alone.

## 3. Neoadjuvant Chemoradiation Therapy

nCRT, or trimodality therapy, was introduced with the understanding that CT treats systemic disease, whereas RT improves local control. In 1996, Walsh et al. ushered in the nCRT era as he delineated the role of induction CRT in esophageal cancer treatment. This landmark paper demonstrated a 37% survival rate among patients treated with nCRT and surgery compared to 7% among those treated with surgery alone [21]. The landmark CROSS trial subsequently reported a pCR rate of 30% and 50% in patients with adenocarcinoma and squamous cell carcinoma following nCRT, respectively [22,23].

According to a recent meta-analysis (2020) conducted by Kumar et al., encompassing 12 randomized controlled trials (n = 2776), nCRT is associated with significantly higher disease-free survival at 5 years (OR = 0.59, 95% CI 0.47–0.74) and OS at 3 years (OR = 0.59, 95% CI 0.47–0.74) compared to surgery alone [24]. However, 5-year OS was not significantly different, and nCRT was associated with a significantly higher perioperative mortality compared to those undergoing surgery alone (OR = 1.79, 95% CI 1.15–2.80). However, the more recent landmark network meta-analysis by Faron et al. (2023) did demonstrate superiority of nCRT over surgery alone—conferring an absolute 5-year survival benefit of 8–10%, irrespective of histologic sub-type [19].

Importantly, most of these trials commenced over 1–2 decades ago, and survival outcomes are likely to improve with the introduction of conformal radiation techniques and meticulous staging utilizing high-quality imaging modalities.

The optimal chemotherapeutic regimen remains undefined; therefore, flexibility in patient-specific management is important. Historically, a combination of cisplatin and 5-fluorouracil was chosen as the standard radiosensitizing regimen with radiation dose ranging from 30 to 45 Gy [25,26]. Current guidelines, however, prefer carboplatin plus paclitaxel (CROSS regimen) due to better tolerability (grade ≥3 toxicity 52% versus 70%), higher treatment completion rates (92% versus 86%), and ease of administration [8,27]. Fluorouracil and oxaliplatin (FOLFOX regimen) represent an alternative with comparable efficacy and a lower risk of nephrotoxicity, without the need for intensive intravenous hydration, compared with fluorouracil/cisplatin [26,28]. This distinction is particularly relevant in patients with preexisting renal impairment or significant cardiac comorbidities.

Table 2 provides details on landmark trials comparing nCRT versus surgery alone.

## 4. Neoadjuvant Chemoradiation Therapy Versus Neoadjuvant Chemotherapy

Several studies have compared nCT and nCRT followed by surgery. Multiple trials suggest noninferiority between the two approaches; however, nCRT is associated with higher tumor response rates compared to nCT alone [32,33,34]. A meta-analysis (2021) by Han et al., including 5 randomized trials and 15 retrospective studies (n = 4529), demonstrated higher R0 resection and pCR rates with lower local/distant recurrence rates in patients receiving nCRT compared to nCT [3]. While this study reported a higher 3-year OS, the 5-year OS was not significantly different [3]. Similarly, the meta-analysis by Faron et al. (2023), including 4 randomized control trials (n = 497), reported an absolute 5-year survival benefit of 0.9 months for nCRT compared to nCT, although this did not reach statistical significance [19]. The most recent meta-analysis by Fleming et al. (2026), which included 9 randomized trials (published 2009–2024) and 2174 patients, highlighted the influence of histologic subtypes on treatment outcomes [35]. Compared with nCT, nCRT was associated with superior pCR rates (32% versus 9%), lower locoregional recurrence, and improved 3-year OS (OR 1.5) in patients with squamous cell carcinoma. In contrast, for adenocarcinomas, nCRT was only associated with a higher R0 resection rate (OR 2.9), without a significant improvement in pCR rates, locoregional recurrence, or 3-year OS.

The higher pCR rate observed after nCRT likely reflects the synergistic effect of the two treatments. Chemotherapy exerts independent cytotoxic effects but also functions as a radiosensitizer, potentially enhancing nodal sterilization beyond that achieved with radiation alone. The clinical significance of achieving a pCR varies and partially depends on the baseline extent of nodal disease. While surgical resection following nCRT can benefit patients with regional N1 or N2 disease, the magnitude of this benefit is greater when patients achieve a pCR, particularly in those with more extensive nodal involvement.

In 2025, the landmark ESOPEC trial was published, comparing nCRT with the CROSS regimen to nCT with the FLOT regimen in patients with adenocarcinoma (cT1N+ or cT3-4N0-3) with a median follow-up of 4.6 years [36]. FLOT was associated with a significantly better progression-free survival at 3 years (52% versus 35%), although it also carried a higher risk of serious adverse events (47% versus 42%). These findings support the use of FLOT as a preferred perioperative regimen in patients with adenocarcinoma, whereas CROSS is preferred in patients with esophageal squamous cell carcinoma.

Table 3 provides details on landmark trials comparing neCRT vs. nCT alone.

## 5. Neoadjuvant Radiotherapy

Current evidence does not support the use of nRT alone in patients diagnosed with resectable esophageal carcinoma. A Cochrane review (2005) of five randomized controlled trials (n = 1147) revealed that nRT is associated with an 11% relative reduction in the risk of death (95% CI 0.78–1.01) and an absolute survival benefit of 4% at 5 years (*p* = 0.06) compared to surgery alone [40]. Another meta-analysis (2020) failed to show a substantial improvement in 5-year OS following nRT [24]. Despite this, radiation does enhance local control and resection rates, with benefits primarily observed in patients with squamous cell carcinoma [41,42,43]. The comparative outcomes of landmark trials comparing nRT versus surgery alone are outlined in Table 4.

## 6. Neoadjuvant Immunotherapy

Neoadjuvant immunotherapy (nIO) is a novel emerging method of targeting cancer cells; however, the current data on this topic is limited. Currently, adding EGFR, VEGF, or HER2 inhibitors to neoadjuvant therapies have proven ineffective. Several smaller randomized controlled trials investigating the role of nIO have been published to date. A meta-analysis (2020) by Wang et al., including 1 randomized control trial and 19 cohort/retrospective studies (n = 621), demonstrated improved pCR rates in patients receiving nIO [45].

Perhaps the most robust guidance for neoadjuvant/perioperative IO in esophageal cancer pertains to mismatch repair-deficient (dMMR)/microsatellite instability-high (MSI-H) tumors. Two randomized phase II trials have reported excellent outcomes. The NEONIPIGA trial (neoadjuvant nivolumab/ipilimumab followed by adjuvant nivolumab) and the INFINITY trial (neoadjuvant tremelimumab/durvalumab) both demonstrated pCR rates of approximately 58–60% [46,47]. A pooled analysis comparing dual nIO with nCT with FLOT in dMMR/MSI-H adenocarcinomas showed markedly higher pCR rates with dual nIO (62% vs. 4%) [48]. Although dMMR/MSI-H is exceedingly rare in esophageal squamous cell carcinoma, the 2025 NCCN guidelines recommend the same neoadjuvant/perioperative IO approach for both histologic subtypes. Other recommended neoadjuvant/perioperative IO regimens-largely extrapolated from studies involving non-esophageal dMMR/MSI-H solid tumors-include dostarlimab [49] and pembrolizumab [50].

In patients without dMMR/MSI-H tumors, nIO, particularly with immune checkpoint inhibitors like nivolumab, has shown promising results in various solid tumors. Studies exploring the role of adjuvant IO after nCRT are more robust, with encouraging outcomes seen in the CheckMate-577 trial [51] using adjuvant nivolumab (in addition to nCRT with CROSS regimen) and the PIECE trial [52] using S-1, a fluoropyrimidine derivative. The JCOG1804E FRONTiER trial is currently underway to examine the efficacy and safety of neoadjuvant nivolumab in patients undergoing nCT, and it has shown promising preliminary results in patients with squamous cell carcinoma [53,54].

Another key target under study is PD-1 inhibition. Published in 2024, KEYNOTE 585 compared the outcomes of nCT (FLOT regimen) with or without pembrolizumab in patients with resectable gastric and gastroesophageal adenocarcinomas [55]. While the trial failed to demonstrate a difference in survival, pCR rates were higher in patients treated with pembrolizumab (13% versus 2%). While this trial has not changed practice, it revealed a potential benefit for PD-1 inhibitors in patients with PD-L1-high or MSI-H genetic phenotypes.

In 2025, the MATTERHORN trial, comparing the outcomes of nCT (FLOT regimen) with or without durvalumab (PD-L1 inhibitor) in patients with resectable gastric or gastroesophageal adenocarcinoma, published its mid-term results [56]. Addition of neoadjuvant durvalumab was associated with significantly better pCR rates (19% versus 7%), nodal negativity, and event-free survival. While long-term results are pending, this benefit has already led to the recommendation of perioperative (neoadjuvant and adjuvant) durvalumab with FLOT for PD-L1 composite positive score ≥1 or tumor area positivity ≥1% tumors.

Two ongoing phase III trials involving esophageal squamous cell carcinoma also warrant attention. An interim analysis of the HCHTOG1909 randomized trial, which compares nCT with and without perioperative toripalimab, demonstrated improved pCR rates with toripalimab (19% versus 4.6%) and 1-year OS (94% versus 83%), with comparable safety profiles [57]. In addition, the prospective multicenter SCIENCE study (NCT05244798) is evaluating nCT or nCRT with sintilimab to nCRT alone [58]. Although phase III data are pending, multiple phase II trials and real-world studies have reported encouraging outcomes [59,60].

Table 5 provides details of landmark trials comparing nIO with nCRT or nCT.

## 7. Conclusions

Neoadjuvant therapy constitutes an essential element in the management of locally advanced resectable esophageal cancer. nCRT for squamous cell carcinoma and perioperative CT with IO for adenocarcinoma, followed by surgery, are now the standard of care in the United States, although this approach is not universally adopted. Not all patients are candidates for concurrent CRT, and these patients benefit from nCT. However, no survival benefit has been demonstrated for nRT alone.

When choosing between nCT and nCRT, toxicity profiles and patient selection should guide decision-making. nCRT is generally associated with higher rates of high-grade adverse events, including cardiovascular complications, chylothorax, or radiation-related esophagitis [39,64]. In contrast, nCT alone is associated with neutropenia and gastrointestinal toxicities and may be poorly tolerated by medically frail patients, particularly when triple-agent regimens such as FLOT are used [36]. Interestingly, a Finnish nationwide study found no significant difference in overall postoperative complications or major adverse events between nCRT and nCT after adjusting for confounders, suggesting that careful patient selection and institutional experience may be as important as the choice of neoadjuvant modality [65].

In this context, tumor burden, performance status, comorbidities, and anticipated toxicity profiles must all be considered. For instance, in patients with borderline resectable or bulky tumors, nCRT may be favored to maximize R0 resection [66]. On the other hand, a medically unfit patient without access to close toxicity monitoring who presents with adenocarcinoma may not tolerate FLOT and could instead derive greater benefit from nCRT using a doublet regimen.

Future studies will help define variables such as the optimal CT regimen, radiation field design, total radiation dose, and the need for surgery following pCR. For now, and the foreseeable future, neoadjuvant therapy followed by surgical resection will remain a mainstay in the treatment of esophageal cancer.

Moving forward, the role of induction therapy will likely focus on more selected patients due to genetic sequencing of tumors or possibly advancements in tumor staging. Perhaps, the biggest frontier will be the integration of IO into treatment strategies. Progress here will be slow as phase 1 trials have just commenced with induction CRT and IO. Although tumor biology differs, recent advances in lung cancer and other malignancies suggest that nCT combined with the unprecedented success of nIO may dramatically change the future management of esophageal cancer.

*Closing Points*

- Neoadjuvant therapy is essential in improving outcomes of patients with locally advanced resectable esophageal cancer.
- nCRT and nCT with IO are standard modalities for locally advanced esophageal squamous cell carcinomas and adenocarcinomas, respectively.
- Future research must determine the optimal regimen, the dose of radiation/chemotherapy, surgical intervals, and better identify patients with a higher risk of toxicity, incomplete pathologic response, and recurrence.

## Figures and Tables

**Table 1:** Clinical trials comparing chemotherapy followed by surgery vs. surgery alone.

Study (Year)	Histology	CT Regimen		Sample Size		Survival (%)			Conclusions
		Cycle	Type	CT	S	Year	CT	S	
RTOG8911 American Trial: Kelson (1998, 2007) [9,10]	Adenocarcinoma and SCC	3	CF	213	227	3	23	26	Improved R1 resection (4% vs. 15%). No difference in OS, local/distant recurrence, R0 resection, or outcomes comparing adenocarcinoma vs. SCC.
Italian Trial: Ancona (2001) [18]	SCC	2–3	CF	47	47	5 *	34	22	Improved 5-year OS in CT responders (complete > partial) vs. non-responders vs. surgery alone (60% vs. 12% vs. 26%). No difference in R0/R1 resection or operative mortality.
OEO2 British Trial: MRC (2002, 2009) [12,20]	Adenocarcinoma and SCC	2	CF	401	401	5 *	23	17	Improved R0 resection (60% vs. 54%) and OS. No difference in distant recurrence rates or outcomes comparing adenocarcinoma vs. SCC. May be confounded by option to give nRT.
MAGIC Trial: Cunningham (2006) [13]	Adenocarcinoma (stomach or GEJ)	3	ECF	250	253	5 *	36	23	Improved OS and DFS. No difference in operative mortality.
EORTC 40,954 Trial: Schuhmacher (2010) [16]	Adenocarcinoma (stomach or GEJ)	3	CF	72	72	2	73	70	Improved R0 resection (82% vs. 67%) and lymph node metastasis (61% vs. 77%). No significant difference in OS.
Dutch Trial: Boonstra (2011) [17]	SCC	2-4	EC	85	84	5 *	26	17	Improved 5-year OS and DFS. No difference in R0 resection.
FNCLCC/FFCD 9703 French Trial: Ychou (2011) [14]	Adenocarcinoma	2–3	CF	113	111	5 *	38	24	Improved OS, DFS (34% vs. 19%), and R0 resection (84% vs. 73%).

* Indicates statistical significance (*p* < 0.05). C, cisplatin; CT, chemotherapy; DFS, disease-free survival; E, epirubicin; F, 5-fluorouracil; GEJ, gastroesophageal junction; OS, overall survival; S, surgery; SCC, squamous cell carcinoma.

**Table 2:** Clinical trials comparing chemoradiation therapy followed by surgery vs. surgery alone.

Study (Year)	Histology	RT Regimen		CT Regimen		Sample Size		Survival (%)			Conclusions
		Gy	Fractions	Cycles	Type	CRT	S	Year	CRT	S	
Irish Trial: Walsh (1996) [21]	Adenocarcinoma	40	15	2	CF	58	55	3 *	32	6	Improved OS and lymph node involvement (42% vs. 82%). No difference in operative mortality. pCR: 25%
French trial: Bosset (1997) [29]	SCC	37	5	2	C	143	139	5	~26	~26	Improved DFS and recurrence rates. Increased operative mortality (12% vs. 3.6%). pCR: 26%
CALBG9781 American Trial: Tepper (2008) [30]	Adenocarcinoma and SCC	50.4	28	2	CF	30	26	5 *	39	16	Improved OS. Early trial termination due to poor accrual. pCR: 40%
CROSS Dutch Trial: Van Hagen (2012, 2015) [22,23]	Adenocarcinoma and SCC	41.4	23	5	CaP	178	188	5 * 10 *	47 38	34 25	Improved OS and R0 resection (92% vs. 69%). No difference in operative mortality. Delayed surgery > 45 days from RT improved pCR. pCR: 29% (SCC: 49% > adenocarcinoma: 23%)
NEOCRTEC5010: Yang (2018) [31]	SCC	40	20	2	CV	224	227	5 *	55.9	49.1	Improved OS and R0 resection (98.4% vs. 91.2%) and DFS. No significant difference in operative mortality. pCR: 43.2%

* Indicates statistical significance (*p* < 0.05). C, cisplatin; Ca, carboplatin; CRT, chemoradiation therapy; CT, chemotherapy; DFS, disease-free survival; E, epirubicin; F, 5-fluorouracil; GEJ, gastroesophageal junction; Gy, gray; OS, overall survival; P, paclitaxel; pCR, pathologic complete response; RT, radiotherapy; S, surgery; SCC, squamous cell carcinoma; V, vinorelbine.

**Table 3:** Clinical trials comparing chemoradiation therapy followed by surgery vs. chemotherapy followed by surgery.

Study (Year)	Histology	Regimen		Sample Size		Significance
		CRT	CT	CRT	CT	
NeoRes Trial: Klevebro (2016) [33]	Adenocarcinoma and SCC	3 cycles CF + 40 Gy	3 cycles CF	90	91	Improved pCR (28% vs. 9%), R0 resection (87% vs. 74%), lymph node involvement (35% vs. 62%). No difference in 3-year OS (47% vs. 49%) or progression-free survival (44% vs. 44%). Trial was not powered to detect subtype specific differences.
POET German Trial: Stahl (2017) [34]	Adenocarcinoma	15 cycles CF + 30 Gy + 1 cycle CE	15 cycles CF	45	48	Improved pCR (16% vs. 1.0%), lymph node involvement (36% vs. 63%), local recurrence (21% vs. 38%), and local tumor progression. No difference in 5-year OS (40% vs. 24%), operative mortality (10% vs. 4%), R0 resection, or distant recurrence (29% vs. 44%).
TOPGEAR: Leong (2017, 2024) [37,38] *	Adenocarcinoma (stomach or GEJ)	MAGIC/FLOT (2 cycles ECF/ECX or 3 cycles of DOF + 45 Gy	MAGIC/FLOT (3 cycles ECF/ECX or 4 cycles DOF)	376	376	Improved pCR (17% vs. 8%) and nodal negativity. No difference in 5-year OS (~45%) and median progression free survival. Note: >70% had gastric cancer.
JCOG1109 NExT Trial: Kato (2022, 2024) [25,39]	SCC	2 cycles CF + 41.4 Gy	2 cycles CF or 3 cycles DCF	200	401	Improved pCR (39% vs. 19%). No difference in OS.
Neo-AEGIS: Reynolds (2023) [32] *	Adenocarcinoma	CROSS (5 cycles CaP + 41.4 Gy)	MAGIC/FLOT (3 cycles ECF/ECX or 4 cycles DOF)	178	184	Improved pCR (16% vs. 5%), R0 resection (95% vs. 82%), lymph node involvement (40% vs. 65%), and tumor regression (42% vs. 12%). No difference in 3-year OS (56% vs. 57%), recurrence, or operative mortality Trial discontinued due to similar survival metrics and COVID-19.
ESOPEC: Hoeppner (2025) [36]	Adenocarcinoma	CROSS (5 cycles CaP + 41.4 Gy)	FLOT (4 cycles DOF)	217	221	Improved pCR (19% vs. 14%), 3-year progression free survival (52% vs. 35%), and 3-year OS (57% vs. 51%) with CT.

* MAGIC regimen before 2018 (ECF or ECX) or FLOT regimen after 2018 (DOF). C, cisplatin; Ca, carboplatin; CRT, chemoradiation therapy; CT, chemotherapy; D, docetaxel; E, epirubicin; F, 5-fluorouracil; GEJ, gastroesophageal junction; Gy, gray; O, oxaliplatin; OS, overall survival; P, paclitaxel; pCR, pathologic complete response; S, surgery; SCC, squamous cell carcinoma; X, capecitabine.

**Table 4:** Clinical trials comparing radiotherapy followed by surgery vs. surgery alone.

Study (Year)	Histology	Radiation		Sample Size		Survival (%)			Conclusions
		Gy	Fraction	RT	S	Year	RT	S	
EORTC: Gignoux (1988) [41]	SCC	33	10	116	113	5	10	9	Improved local failure (46% vs. 67%). No difference in resectability.
Wang (1989) [42]	SCC	40	10	104	102	5	35	30	Improved local failure (34% vs. 41%) No difference in resectability.
Scandinavian Trial: Nygaard (1992) [44]	SCC	35	20	58	50	Not applicable	-	-	No difference in median OS (~7 months for both)

Gy, gray; OS, overall survival; RT, radiotherapy; S, surgery; SCC, squamous cell carcinoma.

**Table 5:** Clinical trials comparing immunotherapy with chemotherapy or chemoradiation therapy followed by surgery.

Study (Year)	Histology	IO	RT Regimen		CT Regimen		Sample Size		Conclusions
			Gy	Fractions	Cycles	Type	+IO	−IO	
SAKK75/08 Trial: Ruhstaller (2018, 2022) [61,62]	Adenocarcinoma and SCC	Cetuximab	45	25	2	CD	149	151	Improved local recurrence (21% vs. 39%). No difference in 4-year OS (56% vs. 43%), operative mortality, or distant recurrence. Trial did not report subtype specific outcomes, however, a subsequent analysis revealed higher complications (65% vs. 51%), particularly acute respiratory distress syndrome (14% vs. 2%) for SCC.
RTOG1010 Trial: Safran (2022) [63]	Adenocarcinoma (HER2+)	Trastuzumab	50.4	28	6	CaP	102	101	No difference in perioperative outcomes or OS.
JCOG1804E FRONTiER Trial: Matsuda (2022, 2024) [53,54]	SCC	Nivolumab	-		CF or DCF		6	6	Phase II. Results pending.
KEYNOTE 585 Trial: Shitara (2024) [55]	Adenocarcinoma	Pembrolizumab	-		4	DOF	402	402	Improved pCR (13% vs. 2%). Median OS was better, but not statistically significant (61 months vs. 58 months).
MATTERHORN Trial: Janjigian (2025) [56]	Adenocarcinoma	Durvalumab	-		4	DOF	474	474	Improved pCR (19% vs. 7%), nodal negativity (58% vs. 45%), and 2-year OS (67% vs. 59%).

C, cisplatin; Ca, carboplatin; CT, chemotherapy; D, docetaxel; F, 5-fluorouracil; Gy, gray; IO, immunotherapy; O, oxaliplatin; OS, overall survival; P, paclitaxel; pCR, pathologic complete response; RT, radiotherapy; SCC, squamous cell carcinoma.

## Data Availability

Data are contained within the article.
